# Increased EZH2 expression in prostate cancer is associated with metastatic recurrence following external beam radiotherapy

**DOI:** 10.1002/pros.23817

**Published:** 2019-05-18

**Authors:** Xiaoning Wu, Helen Scott, Sigrid V. Carlsson, Daniel D. Sjoberg, Lucia Cerundolo, Hans Lilja, Remko Prevo, Guillaume Rieunier, Valentine Macaulay, Geoffrey S. Higgins, Clare L. Verrill, Alastair D. Lamb, Vincent T. Cunliffe, Chas Bountra, Freddie C. Hamdy, Richard J. Bryant

**Affiliations:** ^1^ Department of Oncology, CRUK/MRC Oxford Institute for Radiation Oncology University of Oxford Oxford United Kingdom; ^2^ Nuffield Department of Surgical Sciences University of Oxford Oxford United Kingdom; ^3^ Department of Epidemiology & Biostatistics Memorial Sloan Kettering Cancer Center New York New York; ^4^ Urology Service at the Department of Surgery Memorial Sloan Kettering Cancer Center New York New York; ^5^ Department of Urology Institute of Clinical Sciences, Sahlgrenska Academy at Gothenburg University Gothenburg Sweden; ^6^ Department of Laboratory Medicine, Surgery (Urology), and Medicine (GU‐Oncology) Memorial Sloan Kettering Cancer Center New York New York; ^7^ Department of Translational Medicine Lund University Malmö Sweden; ^8^ Department of Oncology University of Oxford Oxford United Kingdom; ^9^ Oxford NIHR Biomedical Research Centre University of Oxford Oxford United Kingdom; ^10^ Department of Biomedical Science University of Sheffield Sheffield United Kingdom; ^11^ Nuffield Department of Medicine, Structural Genomics Consortium University of Oxford Oxford United Kingdom

**Keywords:** enhancer of zeste 2, prostate cancer, radiotherapy

## Abstract

**Background:**

Enhancer of zeste 2 (EZH2) promotes prostate cancer progression. We hypothesized that increased EZH2 expression is associated with postradiotherapy metastatic disease recurrence, and may promote radioresistance.

**Methods:**

EZH2 expression was investigated using immunohistochemistry in diagnostic prostate biopsies of 113 prostate cancer patients treated with radiotherapy with curative intent. Associations between EZH2 expression in malignant and benign tissue in prostate biopsy cores and outcomes were investigated using univariate and multivariate Cox regression analyses. LNCaP and PC3 cell radiosensitivity was investigated using colony formation and γH2AX assays following UNC1999 chemical probe‐mediated EZH2 inhibition.

**Results:**

While there was no significant association between EZH2 expression and biochemical recurrence following radiotherapy, univariate analysis revealed that prostate cancer cytoplasmic and total EZH2 expression were significantly associated with metastasis development postradiotherapy (*P* = 0.034 and *P* = 0.003, respectively). On multivariate analysis, the prostate cancer total EZH2 expression score remained statistically significant (*P* = 0.003), while cytoplasmic EZH2 expression did not reach statistical significance (*P* = 0.053). No association was observed between normal adjacent prostate EZH2 expression and biochemical recurrence or metastasis. LNCaP and PC3 cell treatment with UNC1999 reduced histone H3 lysine 27 tri‐methylation levels. Irradiation of LNCaP or PC3 cells with a single 2 Gy fraction with UNC1999‐mediated EZH2 inhibition resulted in a statistically significant, though modest, reduction in cell colony number for both cell lines. Increased γH2AX foci were observed 24 hours after ionizing irradiation in LNCaP cells, but not in PC3, following UNC1999‐mediated EZH2 inhibition vs controls.

**Conclusions:**

Taken together, these results reveal that high pretreatment EZH2 expression in prostate cancer in diagnostic biopsies is associated with an increased risk of postradiotherapy metastatic disease recurrence, but EZH2 function may only at most play a modest role in promoting prostate cancer cell radioresistance.

## 1 INTRODUCTION

An estimated 164 690 new prostate cancer (PCa) cases were diagnosed in the United States (US) alone in 2018.^1^ Radical treatment options for localized PCa include radical surgery and radical radiotherapy (RT), which have equivalent cure rates at a median follow‐up of 10 years.^2^ Almost half of the men with high‐risk localized PCa currently receive RT with curative intent,^3^ and while concomitant androgen deprivation therapy (ADT)^4^, ^5^ and advances in external beam RT delivery^6^, ^7^ have improved treatment, RT does not cure all patients. In particular, high‐risk localized PCa can recur following RT, with 5‐year disease‐free survival rates of 78% to 94% being reported for RT plus ADT in large series.^4^, ^8^, ^9^, ^10^

The Polycomb Group protein enhancer of zeste 2 (EZH2) promotes PCa development^11^ and is implicated in tumor cell proliferation, invasiveness, metastasis, and progression to a castration‐resistant phenotype.^12^, ^13^, ^14^, ^15^, ^16^, ^17^, ^18^, ^19^, ^20^ EZH2 functions within the Polycomb Repressive Complex 2 (PRC2) in concert with histone deacetylases (HDACs),^21^, ^22^ and catalyzes a transcriptionally repressive histone H3 lysine 27 tri‐methylation signal.^23^, ^24^, ^25^, ^26^ This results in the recruitment of PRC1, heterochromatin formation, DNA methylation, and gene silencing.^23^, ^24^, ^26^, ^27^ Epigenetic regulators mediate resistance to anticancer therapies such as RT through several mechanisms,^28^, ^29^, ^30^ and HDAC inhibitors can increase radiosensitivity in several cancers^31^, ^32^ including PCa.^33^

This study tested the hypothesis that increased EZH2 expression in baseline diagnostic PCa biopsy clinical samples may be associated with subsequent post‐RT disease recurrence, and that inhibition of EZH2 function might increase PCa cell radiosensitivity in vitro. We report that patients whose PCa tumors expressed high levels of EZH2 at baseline experienced an increased risk of metastatic disease relapse following RT. We also observed that in vitro inhibition of EZH2 function in PCa cells resulted in only a modest increase in sensitivity to RT treatment. Taken together, these results suggest that increased EZH2 function in PCa promotes post‐RT metastatic recurrence through mechanisms above and beyond increased intrinsic radioresistance alone.

## 2 MATERIALS AND METHODS

### 2.1 Patient cohort and clinical follow‐up

The study population comprised 113 men with PCa who received external beam radical RT with curative intent in Oxford between 2000 and 2005 (from a database of approximately 800 such PCa patients), and from whom pretreatment prostate biopsy slides were archived and available, and on whom outcome data were ascertained. Anonymous clinical data were available from medical chart review, including age at diagnosis, date of RT treatment, prostate‐specific antigen (PSA) at diagnosis and during follow‐up, initial standard‐of‐care contemporary staging imaging where performed (usually comprising isotope bone scan or computed tomography (CT) scan if high‐risk disease or PSA greater than 20 ng/ml at diagnosis), clinical tumor (cT) stage, biopsy Gleason grade group, and clinical follow‐up data for biochemical recurrence (BCR) and/or distant metastasis. Patients were reviewed in the clinic at least once every 6 months after RT, for a minimum of 3 years. External beam RT was 3D conformal and CT planned, and typically a 55 Gy dose was delivered to the planned target volume in twenty fractions over 4 weeks with neoadjuvant and concurrent ADT as previously described.^34^, ^35^ Assuming α/β ratio for PCa of 1.8 Gy,^36^ this dose/fractionation schedule is equivalent to 65.9 Gy in 2 Gy fractions. RT was administered to all other patients in fractions of 2 Gy. Using follow‐up data including serial PSA monitoring, isotope bone scans, CT scans, magnetic resonance imaging (MRI) and positron emission tomography (PET)/CT scans, patients were assigned to one of three mutually exclusive groups: long‐term remission, BCR, or radiologically confirmed distant metastatic relapse. BCR was defined using the ASTRO‐Phoenix Consensus criteria^37^ as a PSA rise greater than 2 ng/ml above the post‐RT nadir, without evidence of metastatic disease, and if this occurred patients would usually be commenced on ADT, unless contra‐indicated due to competing comorbidity or frailty. Metastatic PCa was defined as bony, visceral, or lymph‐node metastases on follow‐up imaging (isotope bone scan, or MRI, or PET/CT scan), or inferred by a PSA rise to greater than 100 ng/ml. The study had institutional ethical committee approval (ORB ethics 09/H0606/5 + 5), and appropriate checks for patient consent for anonymous use of tissue for research were undertaken.

### 2.2 Immunohistochemistry

Archival diagnostic formalin‐fixed paraffin‐embedded (FFPE) prostate biopsy samples were selected for this study as described previously.^34^, ^35^ Sections were deparaffinized and rehydrated in the standard manner, endogenous peroxidase activity was inactivated using 3% H_2_O_2_ in methanol, blocked with 5% normal goat serum and incubated with a previously validated anti‐EZH2 primary antibody (anti‐EZH2, clone AE25, cat. no. MABE362, 1:1000; Merck Millipore, Watford, UK)^38^ at 4°C overnight. Following the addition of a biotinylated secondary antibody, an avidin/biotin‐based peroxidase solution was added, followed by 3,3′‐diaminobenzidine solution hematoxylin counterstaining. Sections were dehydrated and mounted as standard. Stained PCa biopsy samples were scored by a consultant uropathologist blinded to patient‐ and tumor characteristics, and a malignant epithelium EZH2 expression intensity score was assigned ranging from 0 (no expression) to 3 (maximal expression), which was multiplied by the percentage of stained cells, to yield a total PCa EZH2 expression score (range, 0‐300) for each of “nuclear” and “cytoplasmic” EZH2. A PCa “total” EZH2 expression score was calculated as the sum of nuclear plus cytoplasmic staining (range, 0‐600). Where “normal adjacent benign prostate” tissue was available within the prostate biopsy samples, a benign “nuclear,” “cytoplasmic,” and “total” EZH2 expression score was similarly obtained.

### 2.3 Cell culture and UNC1999 treatment

LNCaP and PC3 human PCa cell lines were purchased from American Type Culture Collection (ATCC, Manassas, VA) and maintained in Roswell Park Memorial Institute (RPMI)‐1640 (Gibco, Fisher Scientific UK Ltd, Loughborough, UK) supplemented with 10% fetal bovine serum (FBS) in 5% CO_2_ at 37°C as previously described.^12^, ^13^ All cell lines were regularly tested for the absence of Mycoplasma and continuously cultured for no more than 3 months. These cells were chosen as they are widely used in in vitro PCa research, and can be maintained and grown at the necessary cell density required for clonogenic assays. Cells were treated with the EZH2 chemical probe inhibitor UNC1999 (0.1, 0.5, 1.0 μM for LNCaP, and 2.0, 4.0, 6.0 μM for PC3) or an equivalent percentage of dimethyl sulfoxide (DMSO) as a solvent‐treated control for the indicated time‐courses.

### 2.4 Immunoblotting

Protein lysates were prepared using radioimmunoprecipitation assay (RIPA) lysis buffer (0.1% sodium dodecyl sulfate, 1% NP‐40, 1 mM ethylenediaminetetraacetic acid (EDTA), 50 mM Tris PH 7.5, 150 mM NaCl, 0.25% deoxycholate) with protease and phosphatase inhibitors (Roche, Welwyn Garden City, UK). Protein concentration was determined using the Pierce bicinchoninic acid assay (BCA) Protein Assay Kit (ThermoFisher Scientific, Hemel Hempstead, UK). Following sodium dodecyl sulfate polyacrylamide gel electrophoresis electrophoresis and subsequent immunoblotting, bound anti‐EZH2 antibody (1:1000), anti‐histone H3 tri‐methyl K27 (1:1000), anti‐β‐tubulin (1:1000), was detected by developing film from Western blot analysis substrate (Promega, Southampton, UK).

### 2.5 Antibodies

The following antibodies were used in this study: anti‐Histone H3 tri‐methyl K27 (ab192985; Abcam, Cambridge, UK), anti‐EZH2 (clone AE25, cat. no. MABE362; Merck Millipore, Watford, UK) and anti‐β‐tubulin (T4026; Sigma‐Aldrich, Gillingham, UK) for immunoblotting; anti‐EZH2 for immunohistochemistry (anti‐EZH2, clone AE25, cat. no. MABE362, 1:1000; Merck Millipore, Watford, UK)^38^; anti‐γH2AX for immunofluorescence (05‐636‐AF555, 1:500; Merck Millipore, Watford, UK).

### 2.6 Colony formation assays and γH2AX immunofluorescence

LNCaP and PC3 cells were treated with UNC1999 inhibitor or DMSO as a negative control for 96 hours, and then lifted, diluted and plated into six‐well plates in triplicate to perform a colony‐formation assay (CFA). Approximately 500 PC3 cells and 6000 LNCaP cells were plated ahead of irradiation in medium containing DMSO control or UNC1999 at different doses. Cells were left for 24 hours at 37°C (5% CO_2_) to settle and adhere, and treatment plates were then irradiated at 2, 4, and 6 Gy using a Caesium‐137 irradiator, Gamma Service: GSR D1; dose rate 1.938 Gy/min. At 24 hours postirradiation, cells were changed to medium without UNC1999. Colonies were grown for 10 to 14 days postirradiation and then stained with crystal violet, and colonies were then counted using a GelCount colony counter (Oxford Optronix, Abingdon, UK). Effects of UNC1999 treatment on the number of surviving colonies at 0, 2, 4, and 6 Gy were compared against DMSO‐treated control cells using a paired *t* test. For γH2AX foci analysis, UNC1999 and DMSO control‐treated cells were irradiated as described above, and then fixed at different time points using 4% paraformaldehyde, and permeabilized by fresh 0.1% Triton X‐100 in 4% fetal calf serum‐containing solution. Cells were probed with an anti‐γH2AX antibody, and foci were imaged using a Zeiss LSM 710 confocal microscope and quantified using ImageJ software (NIH Image, NIH, Bethesda, MD).

### 2.7 Statistical analysis

We sought to assess whether EZH2 expression levels, as determined by immunohistochemistry on pre‐RT prostate biopsy samples, could predict BCR or distant metastasis after RT when added to standard predictors. Firstly, we assessed the univariate association between EZH2 expression levels (nuclear, cytoplasmic, and total [nuclear plus cytoplasmic]) per 100‐unit change, and the outcome (BCR and metastasis respectively), using Cox regression. We then studied the multivariate association, using Cox regression, between EZH2 expression level and the outcomes, adjusting for PSA, cT stage, and biopsy Gleason grade group. Due to the limited number (n = 17) of patients with image‐confirmed metastatic disease during follow‐up it was not feasible to include these covariates in a single model. Therefore, a risk score was created using PSA (cubic splines were used to account for nonlinearity), cT stage (cT1 vs cT2 vs cT3/4), and biopsy Gleason grade group (1 vs 2 vs ≥ 3) to predict BCR after external beam radical RT treatment. The risk score was then utilized for model adjustment. For models where the EZH2 expression score was significantly associated with the outcome on multivariate analysis, the improvement in discrimination (Harrell's c‐index) was reported and corrected for optimism (to attenuate the discrimination estimate slightly, to better estimate the true discrimination) using bootstrap methods.^39^ BCR free‐ and metastasis‐free survival was calculated using Kaplan‐Meier analysis, and patients who did not recur were censored at the date of last clinical follow‐up. All statistical analyses of the clinical cohort and EZH2 expression were performed using Stata 15.0 (StataCorp, College Station, TX).

All statistical tests of in vitro experiment data were performed as two‐tailed *t* tests and differences were considered significant at a *P* < 0.05. All in vitro colony formation assay data are representative of three independent experiments, each being performed in triplicate, and are presented as the mean ± standard error of the mean (SEM) from these multiple repeat experiments.

## 3 RESULTS

### 3.1 Increased EZH2 expression is associated with prostate cancer metastatic disease recurrence following external beam radical radiotherapy

One hundred thirteen patients with available archival FFPE tissue and clinical follow‐up data were identified from a database of approximately eight hundred PCa patients who received external beam RT with curative intent at our institution between 2000 and 2005. Patient and tumor characteristics (for whom no data were missing for multivariate analysis) are described in Table 1. The majority of patients had biopsy Gleason grade group 2 to 3, and cT2‐3, PCa. The median pretreatment PSA value was 13.0 ng/mL (interquartile range [IQR], 7.6‐20.4 ng/mL). Nuclear, cytoplasmic, and total (nuclear + cytoplasmic) EZH2 expression scores for both PCa tissue (N = 113) and “benign normal adjacent prostate tissue” were available in the biopsy cohort (N = 95 of 113 cases), are shown in Table 2. Over a median follow‐up of 7.9 years (IQR, 6.8‐8.4 years) for the entire cohort, 63 of 113 (56%) patients developed any disease recurrence (defined as BCR or metastatic recurrence). 18 of 113 (16%) patients developed definite metastatic disease recurrence, as defined by bone or soft tissue lesions on radionuclide or CT imaging, or a PSA rise to greater than 100 ng/mL.

**Table 1:** Patient cohort characteristics

	N = 113
Median PSA value, ng/mL	13.0 (IQR, 7.6‐20.4)
Gleason grade group, N (%)	
1	26 (23)
2	30 (27)
3	44 (39)
4	7 (6.2)
5	6 (5.3)
Clinical T‐stage, N (%)	
1	30 (27)
2	41 (36)
3	41 (36)
4	1 (0.9)

Abbreviations: IQR, interquartile range; PSA, prostate‐specific antigen.

Baseline patient and tumor characteristics for 113 patients undergoing external beam radiotherapy with curative intent for prostate cancer are outlined.

**Table 2:** Prostate biopsy EZH2 scores

*Prostate cancer* (N = 113)	
Median “nuclear” EZH2 expression score	40 (IQR, 15‐120)
Median “cytoplasmic” EZH2 expression score	140 (IQR, 80‐210)
Median “total” (nuclear + cytoplasmic) EZH2 expression score	230 (IQR, 160‐300)
*“Normal adjacent benign prostate tissue”* (N = 95 of 113)	
Median “nuclear” EZH2 expression score	40 (IQR, 15‐120)
Median “cytoplasmic” EZH2 expression score	0 (IQR, 0‐40)
Median “total” (nuclear + cytoplasmic) EZH2 expression score	80 (IQR, 30‐140)

Baseline EZH2 scores for the malignant areas of prostate biopsies from samples from N = 113 patients, and for the “normal adjacent benign prostate tissue” where available (in N = 95 of 113 patients), are shown.

**Figure 1:**
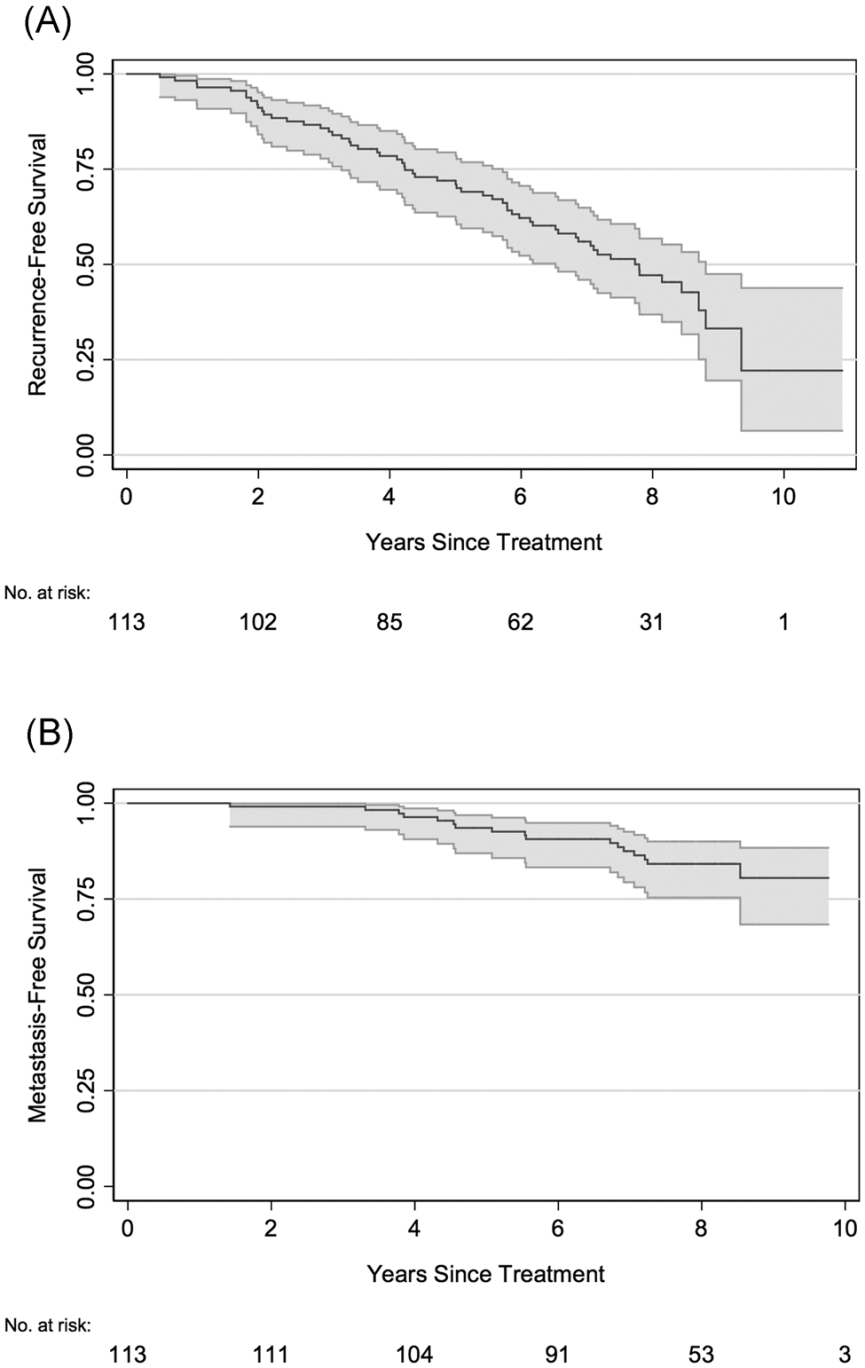
Posttreatment tumor recurrence in the clinical cohort. Biochemical recurrence‐free survival (A) and metastasis‐free survival (B) for the cohort following external beam radical radiotherapy with curative intent

The median follow‐up time for patients who did not develop BCR was 7.8 years (IQR, 6.7‐8.3 years). The 5‐ and 10‐year BCR‐free survival was 72% (95% confidence interval [CI], 63%‐79%) and 22% (95% CI, 6%‐44%), respectively (Figure 1A). No significant association between PCa tissue EZH2 staining levels (nuclear, cytoplasmic, or total) and BCR was observed on either univariate or multivariate analysis (Table 3A).

**Table 3:** Univariate and multivariate analysis of EZH2 expression in the clinical cohort. Univariate and multivariate analyses predicting biochemical recurrence (A) and distant metastasis (B) after external beam radical radiotherapy, based on analysis of the malignant tissue in diagnostic prostate cancer samples. Univariate and multivariate analyses predicting biochemical recurrence (C) and distant metastasis (D) after external beam radical radiotherapy, based on analysis of the “normal adjacent benign prostate tissue” in diagnostic prostate cancer samples were available

	Univariate			Multivariate^a^		
Prostate cancer EZH2 expression	HR	95% CI	*P* value	HR	95% CI	*P* value
*A*						
Nuclear	0.97	0.71‐1.31	0.8	1.07	0.78‐1.45	0.7
Cytoplasmic	1.29	0.98‐1.70	0.069	1.17	0.87‐1.56	0.3
Total	1.24	0.95‐1.61	0.12	1.21	0.92‐1.60	0.2

	Univariate			Multivariate^a^		
Prostate cancer EZH2 expression	HR	95% CI	*P* value	HR	95% CI	*P* value
*B*						
Nuclear	1.30	0.79‐2.13	0.3	1.37	0.83‐2.25	0.2
Cytoplasmic	1.77	1.04‐2.98	**0.034**	1.70	0.99‐2.92	0.053
Total	2.21	1.32‐3.71	**0.003**	2.29	1.32‐3.99	**0.003**

	Univariate			Multivariate^a^		
“Normal adjacent prostate” EZH2 expression	HR	95% CI	*P* value	HR	95% CI	*P* value
*C*						
Nuclear	0.71	0.45‐1.12	0.14	0.78	0.49‐1.24	0.3
Cytoplasmic	1.32	0.75‐2.32	0.3	1.21	0.70‐2.12	0.5
Total	0.85	0.57‐1.26	0.4	0.90	0.60‐1.34	0.6

	Univariate			Multivariate^a^		
“Normal adjacent prostate” EZH2 expression	HR	95% CI	*P* value	HR	95% CI	*P* value
*D*						
Nuclear	0.68	0.27‐1.70	0.4	0.74	0.29‐1.89	0.5
Cytoplasmic	1.01	0.30‐3.40	1.0	0.96	0.29‐3.22	0.9
Total	0.74	0.34‐1.63	0.5	0.78	0.35‐1.74	0.5

Abbreviations: CI, confidence interval; EZH2, enhancer of zeste 2; HR, hazard ratio; PSA, prostate‐specific antigen.

Values in bold represent *P*<0.05.

Hazard ratios are shown for a 100‐unit change in EZH

^a^ Multivariate models adjusted for: PSA, biopsy Gleason score, and clinical T‐stage.

**Figure 2:**
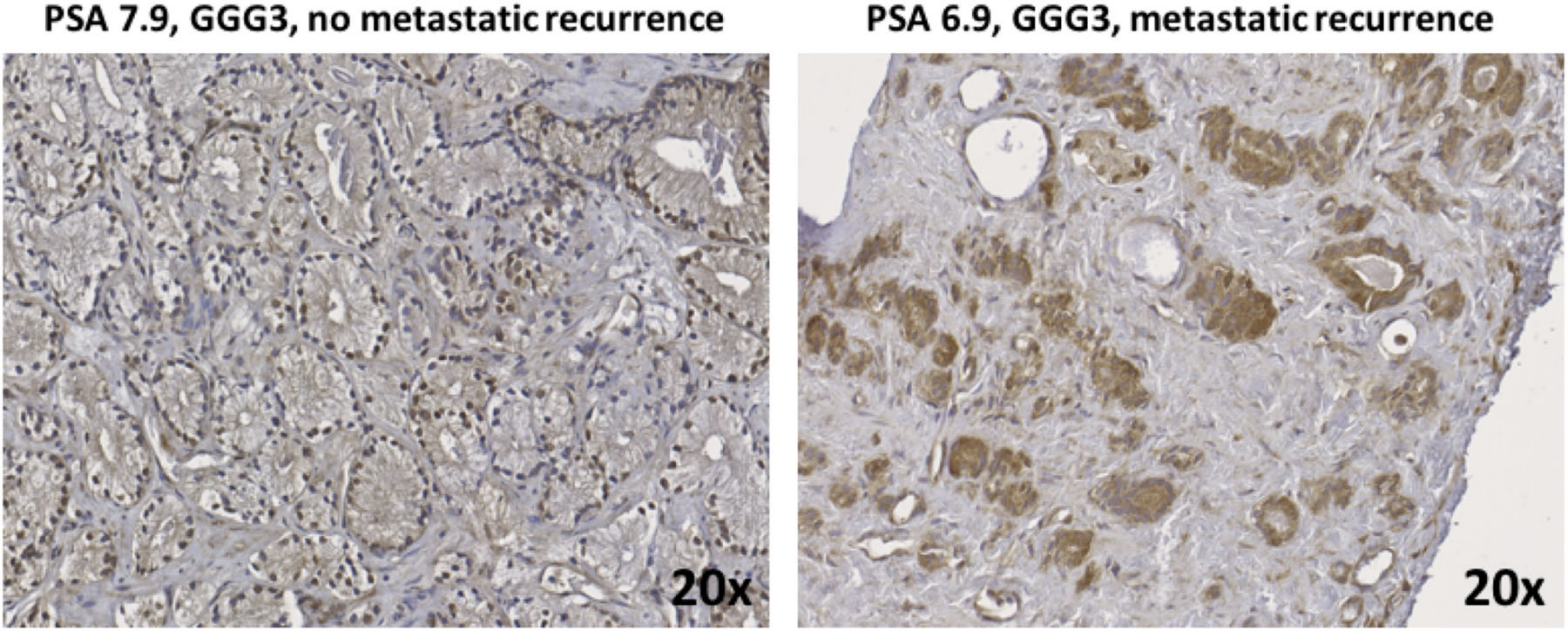
Enhancer of Zeste 2 (EZH2) expression analysis in the prostate cancer cohort treated with radical radiotherapy. Increased EZH2 expression was observed in baseline prostate cancer biopsy samples from individuals with subsequent metastatic progression following radical radiotherapy. PSA, prostate‐specific antigen [Color figure can be viewed at wileyonlinelibrary.com]

The median follow‐up time for patients who did not develop metastasis was 8.1 years (IQR, 7.4‐8.5 years). The 5‐ and 10‐year metastasis‐free survival was 94% (95% CI, 87%‐97%) and 80% (95% CI, 68%‐88%), respectively (Figure 1B). On univariate analysis, PCa tissue cytoplasmic EZH2 expression score, and total (nuclear + cytoplasmic) EZH2 expression score were significantly associated with the development of distant metastasis (*P* = 0.034 and *P* = 0.003, respectively (Table 3B). Figure 2 demonstrates that cytoplasmic EZH2 expression was higher in baseline PCa tissue biopsy samples from patients with subsequent metastatic disease recurrence. On multivariate analysis, the PCa tissue total EZH2 expression score remained significantly associated with metastatic disease recurrence (*P* = 0.003), while the PCa tissue EZH2 cytoplasmic expression score fell marginally short of the conventional level of statistical significance (*P* = 0.053).

The discrimination of the base model (utilizing PSA, Gleason grade group, and cT stage at baseline diagnostic prostate biopsy) for predicting the development of distant metastasis following external beam radical RT was 0.594. After the inclusion of PCa tissue cytoplasmic and total EZH2 expression scores, the optimism‐corrected discrimination estimates for PCa tissue cytoplasmic and total EZH2 expression were 0.676 and 0.723, respectively, representing potentially important improvements in the model's ability to predict the development of posttreatment metastasis following external beam radical RT when PCa tissue EZH2 expression quantification from initial diagnostic biopsies is included.

On univariate and multivariate analysis, “normal adjacent benign prostate” tissue EZH2 expression scores were not significantly associated with the development of either post‐RT BCR or distant metastasis (Tables 3C and 3D).

### 3.2 Investigating the effects of UNC1999‐mediated inhibition of EZH2 function on radiosensitivity of LNCaP prostate cancer cells

**Figure 3:**
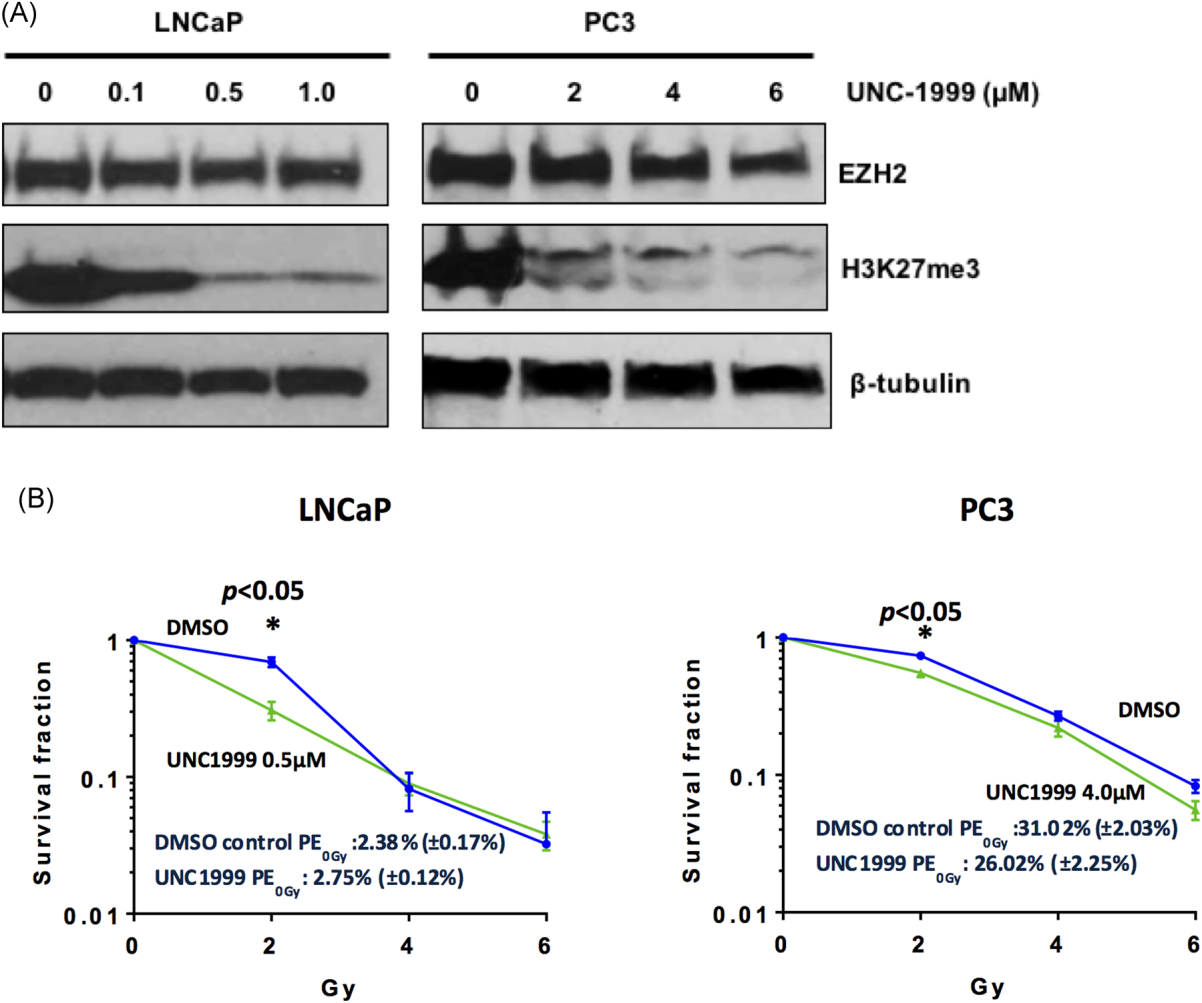
Inhibition of EZH2 function in prostate cancer cells reduced H3K27Me3 levels but only resulted in a modest increase in cellular radio‐sensitivity. H3K27Me3 levels were reduced in LNCaP and PC3 cells following 4 days of treatment with the EZH2‐specific chemical probe UNC1999 (A). 2 Gy irradiation of LNCaP cells in the transient (first 24 hours post radiotherapy) presence of 0.5 μM UNC1999‐mediated EZH2 inhibition resulted in significantly fewer surviving cell colonies (*P < 0.05), and 2 Gy irradiation of PC3 cells in the transient presence of 4.0 μM UNC1999 resulted in a significant reduction in surviving cell colonies (*P < 0.05) (B). This effect was only observed with 2 Gy irradiation. Data shown are means ± SEM of three independent experiments each performed in triplicate. DMSO, dimethyl sulfoxide; EZH2, enhancer of zeste 2 [Color figure can be viewed at wileyonlinelibrary.com]

LNCaP and PC3 PCa cells were treated with various concentrations of the chemical probe UNC1999, which inhibits EZH2 function. As demonstrated by immunoblotting of whole cell lysate preparations, steady‐state H3K27Me3 levels were reduced in both LNCaP and PC3 cells following 4 days of treatment with UNC1999 (Figure 3A), confirming that UNC1999 inhibits the histone methyl‐transferase function of EZH2. Given that inhibition of EZH2 inhibits cellular proliferation^12^ which itself would preclude colony formation in vitro, the lowest doses of UNC1999 with demonstrable inhibition of EZH2‐mediated H3K27Me3 (0.5 µM for LNCaP, and 4.0 μM for PC3) were taken forward for radiosensitivity experiments.

LNCaP and PC3 human PCa cells were treated with ionizing irradiation in the presence of UNC1999, to investigate any potential radiosensitizing effects of EZH2 functional inhibition. Treatment of LNCaP cells with 2 Gy ionizing irradiation in the transient (ie, first 24 hours post‐RT) presence of 0.5 μM UNC1999‐mediated EZH2 inhibition resulted in a modest but statistically significant reduced number of surviving LNCaP cell colonies (*P < 0.05) (Figure 3B). Irradiation of PC3 cells with 2 Gy ionizing irradiation in the transient presence of 4.0 μM UNC1999 also resulted in a modest but statistically significant reduced number of surviving cell colonies (*P < 0.05) (Figure 3B).

**Figure 4:**
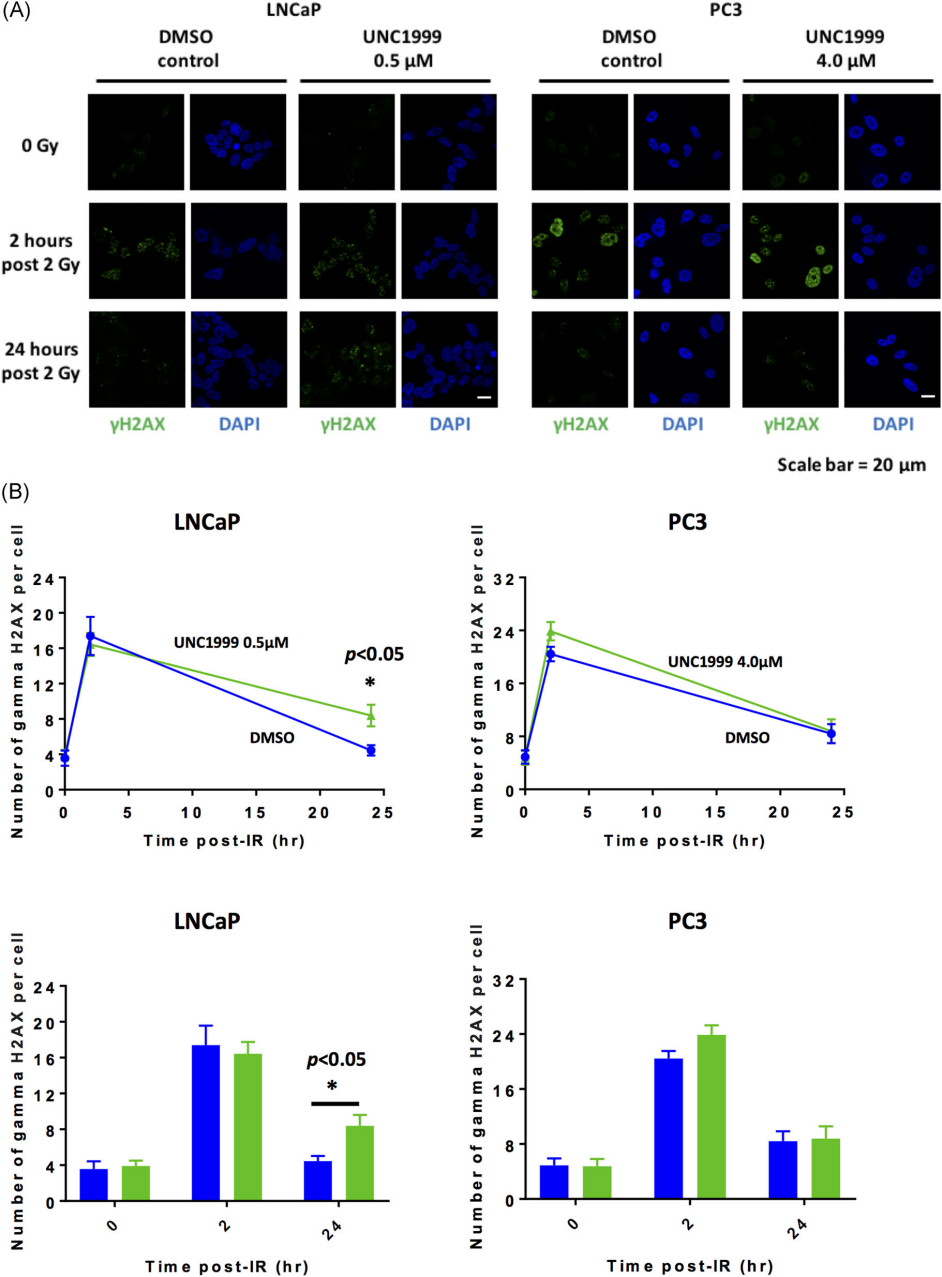
Inhibition of EZH2 function increased γH2AX foci formation in LNCaP, but not PC3, prostate cancer cells following irradiation. γH2AX foci were quantified using immunofluorescence in LNCaP and PC3 cells at 2 and 24 hours following 2 Gy radiotherapy ± UNC1999‐mediated EZH2 inhibition (A). A significantly higher number of γH2AX foci was seen following 0.5 μM UNC1999‐mediated EZH2 inhibition vs DMSO control treated cells at 24 hours postradiotherapy in LNCaP cells (*P < 0.05), but this effect was not observed in PC3 cells treated with 4.0 μM UNC‐1999 (B). Data shown are the representative results of one of two independent experiments and shown as mean ± SEM of γH2AX foci quantified for a minimum of 30 cells in each well. DAPI, 4′,6‐diamidino‐2‐phenylindole; EZH2, enhancer of zeste 2. Abbreviations: DMSO, dimethylsulfoxide; hr, hours; IR, ionizing radiation [Color figure can be viewed at wileyonlinelibrary.com]

γH2AX foci were quantified using immunofluorescence in LNCaP and PC3 cells at 2 and 24 hours following 2 Gy ionizing irradiation ± UNC1999‐mediated EZH2 inhibition. A significantly higher number of γH2AX foci was seen following 0.5 μM UNC1999‐mediated EZH2 inhibition vs DMSO control treated cells at 24 hours postirradiation in LNCaP cells (*P < 0.05) (Figure 4). However, this effect on γH2AX foci formation was not observed in PC3 cells treated with 4.0 μM UNC‐1999.

## 4 DISCUSSION

EZH2 has been implicated in PCa development and progression,^11^, ^12^, ^13^, ^14^, ^15^, ^16^, ^17^, ^18^, ^19^, ^20^ but to date, this knowledge has not yielded clinical benefit for patients. While delivery of external beam RT as a curative treatment option for men with PCa has improved, many patients develop disease recurrence despite concurrent ADT. There is an unmet clinical need to identify druggable targets to increase tumor radiosensitivity,^40^, ^41^ and to identify markers of RT treatment failure. We provide evidence that EZH2 overexpression in pretreatment PCa biopsies is associated with subsequent metastatic PCa recurrence following radical RT.

EZH2 is overexpressed in castration‐resistant PCa, and RT can reduce EZH2 expression in PCa cells.^42^ Moreover, small molecule inhibitors of EZH2 can induce cell death in vitro and in vivo in advanced PCa.^42^ However, studies specifically investigating links between EZH2 expression and PCa recurrence following RT, or the potential for inhibitors of EZH2 to radiosensitize PCa cells, are lacking. Studies in other malignancies suggest that inhibiting EZH2 may enhance RT‐induced inhibition of cancer growth.^43^, ^44^ Non‐small–cell lung cancer studies suggest the efficiency of combined anti‐EZH2 and RT treatment to inhibit cancer cell proliferation differs in various cancer cell lines based on EZH2 expression levels.^43^ We previously demonstrated that LNCaP and PC3 cells express EZH2, and are sensitive to antiproliferative effects of small interfering RNA (siRNA)‐mediated EZH2 inhibition.^12^ While our experiments suggest the radiosensitivity of both LNCaP and PC3 cells can be increased through UNC1999‐mediated inhibition of EZH2 function, this is only a relatively modest effect, suggesting other biological mechanisms may be more significant drivers of metastatic PCa progression post‐RT. Indeed, the effect of combined EZH2 inhibition and RT on γH2AX foci formation was only observed in LNCaP cells treated with 0.5 μM UNC‐1999, and not in PC3 cells treated with 4.0 μM UNC‐1999, suggesting a potential difference in the sensitivity of these cell lines to DNA double‐strand breaks induced by the combined treatment. The p53 status of these two cell lines (PC3 being p53‐null, and LNCaP containing wild‐type p53) might also contribute to the different radio‐sensitivities observed in our experiments, given the important roles of p53 in double‐strand break response and DNA repair.^45^ While further experiments would be required to test this hypothesis, the available evidence suggests that any radiosensitizing effect of EZH2 inhibition in the PCa cell lines tested is modest.

While it is acknowledged that UNC1999 is a selective inhibitor of both EZH2 and EZH1,^46^ we observed the expected reduction in H3K27Me3 levels following treatment of PCa cells with UNC1999. Additional research is necessary to further understand any potential differential oncogenic properties of EZH1 vs EZH2.

While studies investigating potential correlations between baseline EZH2 expression in human cancer samples, and subsequent response to RT, are generally lacking, EZH2 expression correlates with locoregional recurrence in inflammatory breast cancer patients who received RT.^47^ Overexpression of Bmi‐1, a Polycomb Group protein with similar function to EZH2, elicits radioprotective effects through epigenetic effects that counteract the genotoxic insults of RT.^48^ The available in vitro and in vivo evidence, together with our observation that EZH2 expression is associated with metastatic PCa recurrence following RT and only promotes modest radioresistance, support the hypothesis that EZH2 function promotes metastatic recurrence post‐RT, primarily through mechanisms other than increased radioresistance. It remains unknown which of several other downstream functions of EZH2 might primarily account for mechanisms whereby EZH2 promotes metastatic PCa recurrence following RT, however one possibility is that this effect is mediated by increased prostate cancer cellular motility and invasiveness, as this has been demonstrated to be directly promoted by cytoplasmic EZH2 in vitro.^12^, ^49^, ^50^, ^51^ It may be hypothesized that increased cytoplasmic EZH2 function, rather than nuclear function, might promote enhanced PCa cell motility and invasiveness, thereby increasing the risk of developing micrometastases, resulting in enhanced post‐RT disease recurrence. Moreover, if cytoplasmic EZH2 is the main contributor towards the total EZH2 score (the sum of nuclear and cytoplasmic EZH2), then this may explain the observation that cytoplasmic and total EZH2 are associated with metastatic recurrence. It may be the case that the transcriptional repressor nuclear function of EZH2 is not a mechanism underpinning radioresistance, whereas the enhanced cytoplasmic function of EZH2 may promote cellular micrometastasis, leading to post‐RT disease recurrence. Intriguingly, experiments using established radiation‐resistant PCa cell lines demonstrate that they have with higher concomitant cellular motility than parental radiosensitive cell lines.^52^ Further research is necessary to identify the molecular mechanisms underpinning the observed link between cytoplasmic EZH2 expression and post‐RT metastatic recurrence.

Our observation that high levels of EZH2 expression in PCa may promote post‐RT metastatic recurrence has the potential for clinical utility in two main areas. Firstly, given that EZH2 inhibitors have been developed for cancer therapy,^53^ our in vitro data suggest that combining external beam RT and EZH2 inhibitors to treat PCa patients may not result in clinical benefit in terms of radiosensitization per se. However, there is secondly the possibility that patients with high EZH2 expression in baseline samples may benefit from EZH2 inhibition to reduce the risk of metastatic progression. This possibility requires further investigation in larger scale prospective studies.

While the 10‐year BCR‐free survival was low at 22% for patients within this cohort, this is comparable with reported rates of 30% for high‐risk disease following external beam RT,^54^, ^55^ and it is possible that BCR at such a mature length of follow‐up does not equate to true disease recurrence. While it is a strength of our study that the cohort was mature with a median follow‐up of 7.9 (IQR, 6.8‐8.4) years, we acknowledge that these were not consecutive patients from our institution due to inherent constraints acquiring archival tissue with retrospective follow‐up. Indeed, our cohort size of 113 patients is modest, though this has been sufficient to identify other potential mediators of radioresistance.^34^, ^35^ Data on the larger cohort from which these patients originated was unfortunately not available. It would be helpful to validate our findings in similar cohorts from independent institutions.

Patients developing metastatic PCa recurrence following external beam RT may include those with occult micrometastases at the time of irradiation, along with others with local disease recurrence within the radiation field due to radioresistant PCa. Thirty percent of post‐RT BCR is estimated to be due to local recurrence indicative of clinical radioresistance.^56^ A weakness of our study is that it is difficult to differentiate between patients who may have had micrometastases at baseline, and those who may initially have developed local recurrence with a subsequent metastatic phenotype, because post‐RT imaging was generally not performed until BCR occurred, and not all patients in the cohort received re‐staging imaging. A contemporary cohort may receive PSMA‐PET/CT^57^ to accurately evaluate local vs metastatic disease recurrence post‐RT, but as this is only a recent clinical development such a cohort with accurate recurrence classifications would lack long‐term follow‐up. It will be valuable for future studies to investigate whether EZH2 expression in pre‐RT samples predicts post‐RT disease recurrence in cohorts with accurate post‐BCR stage classifications based on molecular imaging.

## 5 CONCLUSIONS

In conclusion, patients with a high level of EZH2 expression in the baseline diagnostic PCa biopsy specimens had an increased risk of metastatic disease recurrence following external beam RT with curative intent. Chemical probe‐mediated inhibition of EZH2 function only results in a modest increase in radiosensitivity of PCa cells in vitro. Taken together this suggests that EZH2 function promotes post‐RT metastatic disease recurrence in PCa patients, and this is likely to be through mechanisms above and beyond any potential increased radio‐resistance mediated by EZH2 function.

## CONFLICT OF INTERESTS

The authors declare that there are no conflict of interests.
